# MSM Book Review

**Published:** 2007

Ever since David Healy first announced in 1991 that SSRIs (selective serotonin reuptake inhibitors) have a causal role in some suicide cases, he has had to face ostracism from corporate psychiatry, has had a job offer from the University of Toronto withdrawn, been accused of practicing ‘junk science’ by the pharmaceutical industry and of covertly promoting a non-SSRI antidepressant via a secret plot with a particular company and of constructing with Carl Elliott his own martyrdom. *The New York Times* (Nov 15, 2005) recently dubbed Healy ‘psychiatry's gadfly’ for his stinging criticism of his profession's blind acceptance of the safety and efficacy data provided by drug companies-data based on their clinical trials of their own drugs.

*Let Them Eat Prozac* details in quasi-autobiographical fashion the events that led up to Healy's discontent with psychiatry's cozy relationship with the pharmaceutical industry. The critical issue of concern is the aggressive marketing of the SSRIs that has usurped the authority of science and led to a cover-up of the risks involved in the use of these psychotropic drugs. The story begins with the case of Caitlin Hurcombe, a 19-year old girl who hanged herself in her bedroom a few weeks after starting to take Prozac. Healy explains, ‘SSRIs can cause suicide in individuals who have no nervous conditions, primarily by inducing mental turmoil during the early stages of treatment. Patients who develop akathisia while on SSRIs are likely to be seen by primary-care physicians who have not been trained to recognize the problem’ (p. 15). The role of akathisia, a drug-induced form of intense agitation, is crucial to linking these drugs with suicide. From the Hurcombe case, we are guided through the history of psychopharmacology in the 20^th^ century, leading up to the creation of the SSRIs, the debate over the effectiveness of these drugs and the adverse reactions they cause and the predictable response from an industry that collectively makes $10 billion a year from the sales of SSRIs. After discussing in detail the different lawsuits that have been filed in the United States alleging a link between SSRI use and suicide or murder, the book ends with the Toronto affair, where the offer of a university post for Healy was withdrawn after he suggested in a lecture there that the SSRIs could cause suicidal behavior.

According to Healy, we have been sold the idea that depression is caused by a chemical imbalance in the brain not because it is the view held by the leading neuroscientists of the world but, rather, because it is a convenient marketing tool for the pharmaceutical industry. The crucial work on serotonin began in the 1960s when researchers found lowered levels of the main serotonin metabolite, 5-hydroxyindole acetic acid, in the cerebrospinal fluid of depressed subjects. By 1970, however, the very individuals who had created the serotonin hypothesis abandoned it. As Healy writes, ‘more sensitive studies had shown no lowering of serotonin in depression. Indeed, no abnormality of serotonin metabolism in depression has ever been demonstrated’ (p.12). Nonetheless, in a victory of marketing over science, the simplistic idea that SSRIs can be used as a kind of dipstick to check the serotonin levels in our brains and to restore us to normality has stuck. Healy attributes this to a widespread use of a vacuous ‘biobabble’ that has replaced the ‘psychobabble’ of the Freudian world view (p. 264).

Contrary to the message of Peter Kramer's 1993 bestseller, *Listening to Prozac*, Healy issues a warning, advising us to weigh the relative risks against the benefits of the SSRIs. Where Kramer's clinical experience of a handful of cases provided a basis for great optimism in treating depression and for explorations in self-discovery induced by Prozac's ‘better-than-well’ feeling, Healy expresses alarm at the very idea that we are being sold lifestyle options with blockbuster pharmacology rather than serious medicine. In 1993, the public was unaware of agitation, akathisia, drug-induced suicidality, aggression and the risks of long-term chemical dependence that could be caused by SSRIs, but the pharmaceutical industry knew, from clinical trials and adverse-events reporting systems, that there were problems. Since, however, the pharmaceutical companies own the data of the clinical trials, many of the failures of these drugs never see the light of day. Healy writes: ‘I now believe it was unreasonably dangerous to leave Prozac, Zoloft, Celexa and Paxil on the market without warnings. I believe there has been a failure by regulators and experts in psychiatry to resolve a problem to which students of medicine could have given the correct answer’ (p. xvi). The warnings he has in mind are not the ones regarding the relatively mild somatic side effects that are mentioned on the labels but, rather, the serious ones that the drug companies have worked hard to keep out of the labels.

Healy squarely faces the question of the degree to which psychiatrists themselves are to blame for this state of affairs. In the preface, he suggests that the ‘academics who were the substantive movers in health-care developments are now serving as ornamental additions to business’ (p. xv). It is the clinicians who are responsible for containing the pharmaceutical industry, but their professional integrity has been corrupted by self-interest. He writes: ‘it is harder to defend the deafening silence of psychiatrists faced with published clinical-trial evidence that the relative risk of suicidality from the newer antidepressants is greater than that from placebo’ (p. 275). In addition to the money paid for their services as ‘key opinion leaders’ and for their work as primary investigators in clinical trials, corporate psychiatrists own substantial stocks in the companies whose drugs they prescribe. The epidemic of depression translates into huge profits for psychiatrists, who are largely unwilling to admit a conflict of interest. Healy recognizes that his exposure of the deception plays into the hands of antipsychiatry, but he stops short of condemning psychopharmacology. He still believes in the therapeutic value of antidepressants, but argues that they are not for everyone.

There is, however, one question that Healy has not answered as clearly as I had expected. If, as he makes clear from the outset, the serotonin hypothesis of depression has been thoroughly refuted, then why does it remain such an influential theory of depression in psychiatry today? The suggestion that it is merely a vacuous ‘biobabble’ does not quite explain the powerful hold that the paradigm of chemical imbalance continues to exert on the profession. In the fateful lecture at Toronto-Psychopharmacology and the government of the self—he says: ‘The era of depression that we have lived through in the 1990s in the West has arguably been a politically and economically constructed era that bears little relationship to any clinical facts.’ This raises questions about the moral integrity of a profession clinging to an erroneous theoretical construction and opts to play a language game that has rationalized the megadose regimes.

As for the causal connection between SSRIs and suicide, many of Healy's critics have dismissed him as an alarmist who has driven patients away from their much-needed medication. The government regulators and the industry have long maintained that there is no credible evidence linking the drugs to suicide. The controversy over the issue of whether SSRIs increase the risk of suicide continues in the leading medical journals, yet the lack of any consensus comes as no surprise at a time when it is estimated that over 50% of the articles are ghostwritten by the pharmaceutical companies. Recent revelations, however, appear to vindicate Healy's position. First, in 2004 government regulators in the UK and the US issued warnings about the emergence of suicidality, especially at the onset of SSRI therapy. Then the FDA, faced with pressure from an action by UK regulators, followed up with the strongest warning possible-the ‘black box’ warning-that describes the increased risk of suicidality in the pediatric population on SSRIs. Second, the industry's response to ‘the Healy problem’ was a public-relations campaign that was designed to discredit Healy's scientific reputation and the results of his healthy-volunteer study in which two patients on SSRIs became suicidal. Suicidality, they maintained, is always a tragic result of the underlying disorder of depression. Aside from the alleged weaknesses in the design of Healy's small study, the central point is that their claim that suicide is always caused by the underlying disorder and never the drug is contradicted by the results of their own healthy-volunteer studies that have been suppressed by ‘the file-drawer phenomenon’—favourable studies being selected for publication while those that reveal unfavourable results in safety and efficacy are filed away. Third, in a surprising reversal of the decade-long denial of any increased risk of suicidal behavior on SSRIs, GlaxoSmithKline (GSK), the maker of paroxetine (Paxil, Seroxat), in a letter to healthcare professionals in May 2006, advised of a label change and warned that there is a possibility of increased risk of suicide-related behaviour whether the drug is prescribed for depression or for other conditions not associated with suicide. Results of a new meta-analysis of suicidal behaviour and ideation in placebo-controlled clinical trials of paroxetine showed that the frequency of suicidal behaviour in young adults treated with paroxetine was higher and that this may extend beyond the age of 24.

The chronology of events, with the early suspicions about SSRI-induced suicidality, the denial of any such connections by industry and by the vast majority of psychiatrists and the slow process of acceptance by the industry and regulators of the evidence that has been present from the outset has shown that time is on Healy's side. *Let Them Eat Prozac* is a lesson that with the corporatisation of medicine *caveat emptor* remains as relevant now as it was before statutory law offered consumers protection in the quality of goods.

## Postscript

[Author's note: The original review was initially accepted for publication in a prestigious journal. Nine months later the review editor wrote to me to say that he would not be able to publish it. He said that he had re-read the review when it was time to publish and had decided to consult a psychiatrist who was of the opinion that there were sweeping generalisations in the review for which no supporting evidence was given. I complained that the review had already been accepted for publication and that the job of a book reviewer is to give a fair exposition of the book's contents and any relevant critical evaluation, which was what I had done. The editor apologized, saying that it was all his fault for not reading the review carefully the first time, but reminded me that the contract I had signed allowed the journal to decline publication at any time.

The review was then submitted to another prestigious journal and accepted for publication by the review editor. However, 3 months later he wrote to say that their lawyers had advised against publishing the review because the book was judged to be potentially libellous and that, therefore, the review might also invite such an action in England. The lawyers also warned that I could be party to a libel action against the journal if I were to continue with publication. I protested, saying that the review had already appeared in another journal of the group and they and their lawyers were being inconsistent.

The review was then submitted to a third journal, where it underwent a peer review and was very slightly modified at the beginning and at the end to update the reader on the ‘Healy problem’ and the current status of the SSRI-suicide debate. They bravely accepted it knowing its history, but there was a bit of a logjam with many papers in their queue, awaiting publication. The review has been waiting in a queue with the journal for a whole year and that brings us to the present].

## About The Author

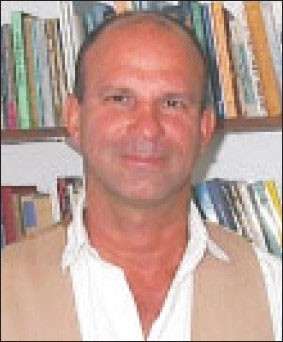



Leemon B McHenry,

California State University, Northridge

*Leemon McHenry read philosophy at the University of Edinburgh where he wrote a PhD thesis on the metaphysics of Alfred North Whitehead and F. H. Bradley. He is currently lecturer at California State University, Northridge, USA and research consultant for the Baum Hedlund law firm of Los Angeles, California. His research interests include metaphysics, philosophy of science and medical ethics*.

